# Micro-nano structure hard carbon as a high performance anode material for sodium-ion batteries

**DOI:** 10.1038/srep35620

**Published:** 2016-10-18

**Authors:** Peng Zheng, Ting Liu, Shouwu Guo

**Affiliations:** 1 School of Materials Science and Engineering, Shaanxi University of Science and Technology, Xian 710021, Shaanxi, P. R. China; 2 Department of Electronic Engineering, School of Electronic Information and Electrical Engineering, Shanghai Jiao Tong University, Shanghai 200240, P. R. China

## Abstract

Superior first-cycle Coulomb efficiency (above 80%) is displayed by filter paper-derived micro-nano structure hard carbon, and it delivers a high reversible capacity of 286 mAh g^−1^ after 100 cycles as the anode for Na-ion battery at 20 mA g^−1^. These advantageous performance characteristics are attributed to the unique micro-nano structure, which reduced the first irreversible capacity loss by limiting the contact between the electrode and electrolyte, and enhanced the capacity by accelerating electron and Na-ion transfer through inter-connected nano-particles and nano-pores, respectively. The good electrochemical performance indicates that this low-cost hard carbon could be a promising anode for Na-ion batteries.

Since first being produced by Sony in 1990, Li-ion batteries (LIBs) have been successfully used in portable electronic equipment owing to their high energy/power density and long lifecycle^1^. With the potential large demand for electric vehicles and large-scale energy storage applications (i.e., solar, wind and geothermal energy), the limited and uneven distribution of lithium resources make exploring new, alternative battery imperative^2^,^3^. Sodium has chemical characteristics similar to lithium but also has a larger radius, higher potential and is naturally abundant worldwide. So, Na-ion batteries (NIBs) have been regarded as a potential alternative to Li-ion batteries^4^,^5^,^6^. Up to now, the reported anodes for NIBs have focused mainly on alloys (e.g., Sb and SnO)^7^,^8^, metal oxides (e.g., Fe_2_O_3_, MnO_2_ and Co_3_O_4_)^9^,^10^,^11^, metal sulfates (e.g., MoS_2_, WS_2_ and FeS_2_)^12^,^13^,^14^ and carbonaceous materials^15^,^16^,^17^,^18^. Because of the merits of natural abundance, low cost, structure stabilization and the successful commercial anode of graphite for LIBs, carbonaceous materials are considered as the most promising option among the above anodes^19^,^20^,^21^. There has been a great deal of research activity on various carbons including hard carbons^22^,^23^,^24^, carbon fiber^25^,^26^,^27^, graphene^28^,^29^,^30^, and carbon spheres^31^,^32^ to improve the performance of NIBs.

In addition to a large capacity, the high first-cycle Coulomb efficiency is also a vital index for a successful anode material. Large surface area carbonaceous materials such as meso-carbon and graphene based anodes can deliver high capacity and good rate performance owing to the broad contact between electrolyte and electrode, which facilitates the fast transfer of electrons and Na ions^33^,^34^,^35^,^36^. However, their capacities after the first cycle significantly decreased owing to increased side reactions with the electrolyte to form a solid-electrolyte interphase (SEI) layer and irreversibly trapped Na ions in the graphene layers and defect sites; these effects are magnified as the surface area increased^37^,^38^,^39^. Up to now, there have been few reports that the first-cycle Coulomb efficiency can exceed 75% of carbon-based anodes with high capacity for NIBs, which would have a severe negative effect on the performance for the Na full cell^40^,^41^,^42^. Micrometer-sized materials with low surface area often have high first-cycle Coulomb efficiency, but the long diffusion path for electrons and Na ions results in poor capacity^43^. To address this deficiency, incorporating a micro-nano structure is an effective strategy^44^. Owing to its special morphology, it can avoid the disadvantages described above to show the superiority of nano- and micro- structure. Micro-nano structure has exhibited superiority in LIBs^45^,^46^,^47^. For example, Yang *et al*.^48^ synthesized a Co_3_O_4_ micro/nano-structure with the first-cycle Coulomb efficiency of 73.8% that shows a high capacity of 970 mAh g^−1^ after 140 cycles as anode materials for LIBs. Lou *et al*.^49^ prepared Co_x_Mn_3-x_O_4_ array micro-/nanostructures with the first-cycle Coulomb efficiency of 57.6%, which have remarkable capacities (540–207 mAh g^−1^) at various current rates (1–10 C) for LIBs. Xie *et al*.^50^ fabricated LiMn_2_O_4_ hollow microspheres composed of nanoparticles that deliver a discharge capacity of 127 mAh g^−1^ at C/10 rate. However, there are few reports on the synthesis of micro-nano structure carbon.

To prepare the special micro-nano structure carbon, our strategy utilizes a chemical etching process, including high temperature pyrolysis and further KOH activation treatment. We chose filter paper as the carbon precursor in part owing to the merits of waste reuse and that the derived carbon fiber is suitable for Na-ion storage^25^,^51^,^52^. The product demonstrates first-cycle Coulomb efficiency as high as 80% and 286 mAh g^−1^ after 100 cycles as the anode for NIBs. The excellent high first-cycle Coulomb efficiency is attributed to the special micro-nano structure of the produced hard carbon, which can reduce the irreversible capacity loss for SEI formation and electrolyte decomposition. To further verify the advantage of the filter paper-derived micro-nano structure carbon with respect to its better performance as an anode for NIBs, a systematic comparison of filter paper-derived micro and nano-sheet carbons was also performed in this study.

## Results and Discussion

**Figure 1:**
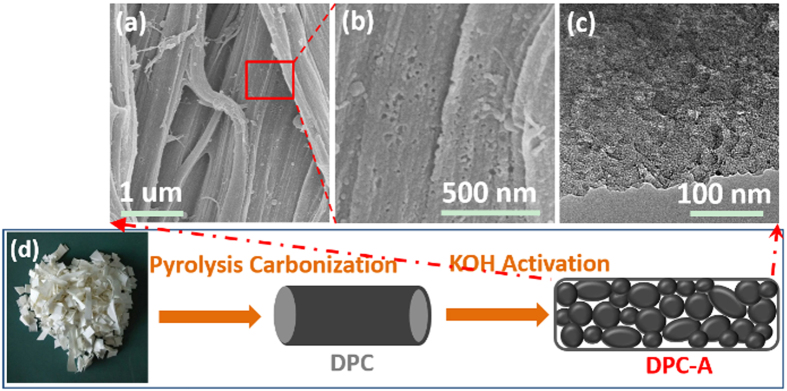
Schematic illustration of the synthetic procedure for filter paper-derived micro-nano structure carbon.

To synthesize micro-nano structure carbon, whole frame reinforcement for micro and local dissolution for nano strategies were employed. Owing to the merit of its large storage of biomass and three-dimensional, highly cross-linked polyphenolic polymer structure, we choose filter paper as the precursor; the fabrication procedure is schematically depicted in Fig. 1d. The first process was high temperature pyrolysis at 800 °C under Ar atmosphere. During the carbonization and reinforcement course, oxygen and hydrogen were removed and the micro-fiber (denoted as DPC) was produced without being crushed (Fig. S1). Subsequently, DPC was further etched by 7 M KOH to local dissolution for formation of nano-particles and nano-pores; the resultant product was denoted as DPC-A. Scanning electron microscopy (SEM) and transmission electron microscopy (TEM) images clearly reveal the micro-nano structure of DPC-A. As shown in Fig. 1a, DPC-A keeps the morphology of coarse fibers that are composed of nano-particles with a size of approximately 20 nm (Fig. 1b,c). The integral connected micron-fiber structure favors electron transfer. The nano-pores among the nano-particles could also be observed clearly, which are beneficial for storage and transfer of Na ions.

**Figure 2:**
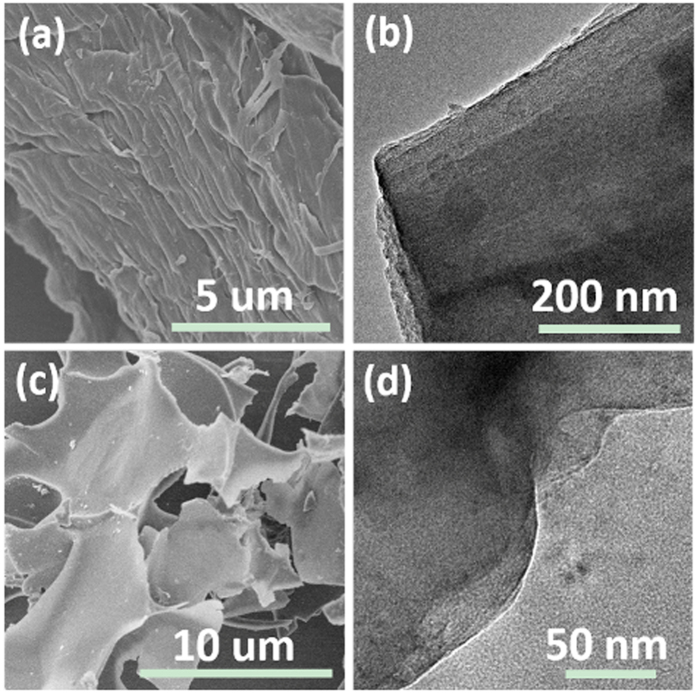
(**a,c**) SEM images of DPC and A-DPC, respectively; (**b,d**) TEM images of DPC and A-DPC, respectively.

**Figure 3:**
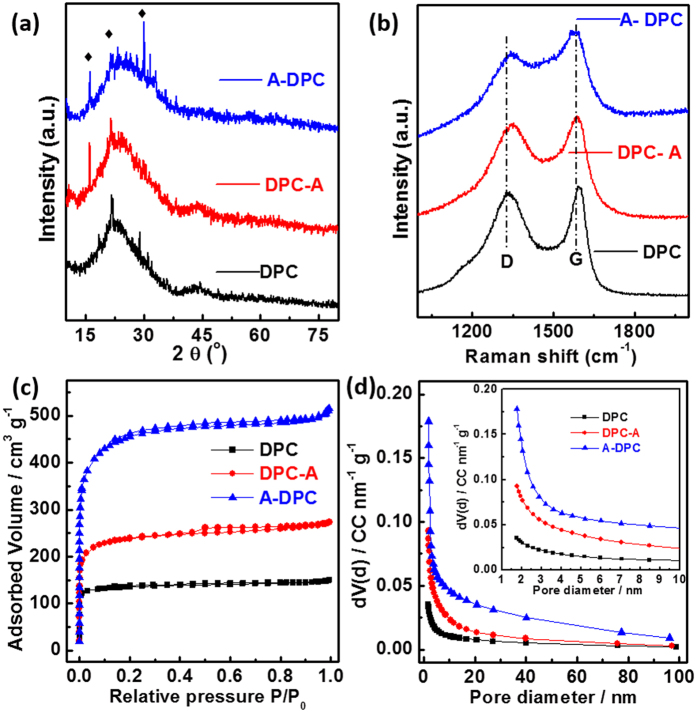
(**a**) XRD patterns; (**b**) Raman spectra; (**c**) N_2_ adsorption–desorption isotherms and (**d**) pore size distribution curves for filter paper-derived carbons.

As shown in Fig. 2a,b, the direct high temperature pyrolysis carbon (DPC) is a micro-fiber that is agglomerated in a similar fashion as the original filter paper (Fig. S2). No nano-pores are observed for DPC and the surface area of DPC is smaller than that of DPC-A (Fig. 3c). To systematically investigate the effect of surface area on the first-cycle Coulomb efficiency, the sample with a larger surface area than that of DPC-A was also prepared (denoted as A-DPC) (Fig. 2c). The detailed synthetic procedure is found in the experimental section. Figure 2(c,d) shows the morphology of A-DPC; it is a nano-sheet with a smooth surface area. For the DPC-A sample, after the first high temperature pyrolysis process, the product has the stable framework; when treated by KOH, only etching took place, so the nano-pores are produced. The etching and fusion processes both occurred for the A-DPC sample, as the original paper is much softer than DPC, so nano-sheets are formed^53^.

Figure 3 shows the structure of three types of filter paper-derived carbons. Figure 3a displays the XRD patterns of the as-prepared carbons; two broad peaks at approximately 2θ = 23° and 44° were assigned to the two typical planes of (002) and (101). Filter paper derived carbons are amorphous carbon and the (101) peak for A-DPC is almost gone. The asterisk-marked peaks are impurities of potassium carbonate crystals, which are indicated in Figure S4d. Figure 3b shows the characteristic D band (defect and disordered carbon) and G band (graphitic carbon) for the derived carbons. The ID/IG ratios were 0.937, 0.936 and 0.841 for DPC, DPC-A and A-DPC, respectively. The lowest ratio implies that A-DPC has the lowest defect among the samples. As indicated by the investigation of X-ray diffraction (XRD) patterns and Raman spectra, filter paper derived carbons are all hard carbons. The detailed surface area and pore structure could be detected by N_2_ adsorption-desorption isothermal analysis (Fig. 3c,d). All of the derived carbons exhibit a type-I/IV isotherm with a sharp adsorption knee at low pressures and well-developed plateaus. In addition to the micropore sorption at low pressure for DPC, the type H4 hysteresis loop indicates the presence of mesopores for DPC-A and A-DPC. The specific surface areas based on the standard Brunauer–Ennett–Teller (BET) method are 431, 761 and 1472 m^2^ g^−1^ for DPC, DPC-A and A-DPC, respectively. As revealed by the pore size distribution curves of Fig. 3d, DPC-A and A-DPC have few mesopores. Combining the XRD, Raman and BET analyses, the derived hard carbon of DPC-A has large interlayer spacing, rich pores and moderate surface area; these characteristics favor Na-ion reversible storage.

**Figure 4:**
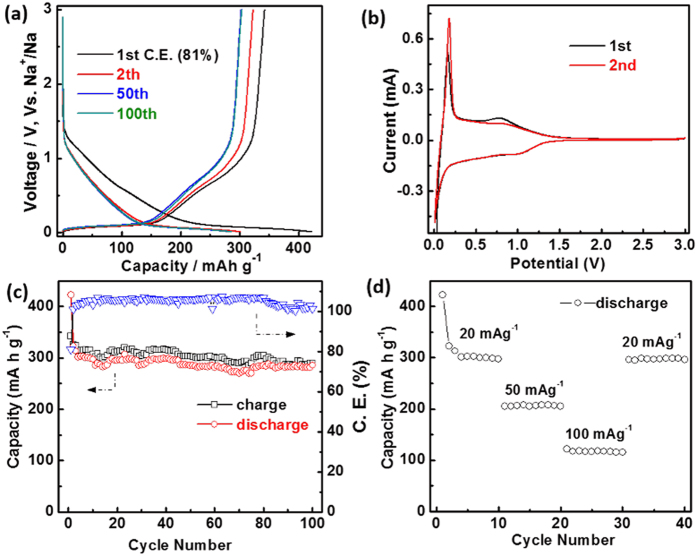
Electrochemical performance of DPC-A electrode: (**a**) charge-discharge voltage profiles at a current density of 20 mA g^−1^; (**b**) representative CV at a scan rate of 0.1 mV s^−1^; (**c**) cycling performance and Coulombic efficiency at current densities of 20 m A g^−1^; (**d**) rate capability at different current densities.

As the anode for NIBs, the filter paper-derived micro-nano structure carbon of DPC-A was tested in the half cell (2032) and its performance is shown in Fig. 4. The DPC-A electrode delivers high discharge capacities of 422, 323, 285 and 286 mAh g^−1^ at 20 mA g^−1^ for the 1^st^, 2^nd^, 50^th^ and 100^th^ cycles, respectively (Fig. 4a). During the first cycle, the Coulomb efficiency (C.E.) can reach as high as 81%. The detailed mechanism was characterized by cyclic voltammetry (CV). The initial two CV curves of the DPC-A electrodes are shown in Fig. 4b in a potential widow of 0.01–3 V at a scan rate of 0.1 mv s^−1^. There is a surplus peak at 0.7 V that occurs between the 1^st^ and 2^nd^ cycles and is attributed to the formation of SEI. As suggested by Ji *et al*.^16^, the broad redox peaks of 0.8 and 1.0 V are regarded as the result of reversible (de)insertion of sodium at defect sites and correspond to the sloped region of the curves. The sharp redox peaks below 0.2 V are considered to be the result of reversible sodium insertion into graphene sheets and minor phenomenon of Na-ion adsorption on pore surfaces, and correspond to the plateau region of the discharge curve. The DPC-A electrode also shows good cycling performance and rate capability. During 100 cycles at a current density of 20 mA g^−1^, the capacities always maintain equal capacity, and at the 100^th^ cycle, the capacity was 286 mAh g^−1^ (Fig. 4c). As the current density was changed to 50 and 100 mA g^−1^, high reversible capacities of 206 and 117 mAh g^−1^ were reached, respectively. The current density was then changed back to 20 mA g^−1^ and the capacity was restored to 289 mAh g^−1^ (Fig. 4d). Such good cycle and ratio properties could be due to the steady frame structure and suitable amounts of pores.

**Figure 5:**
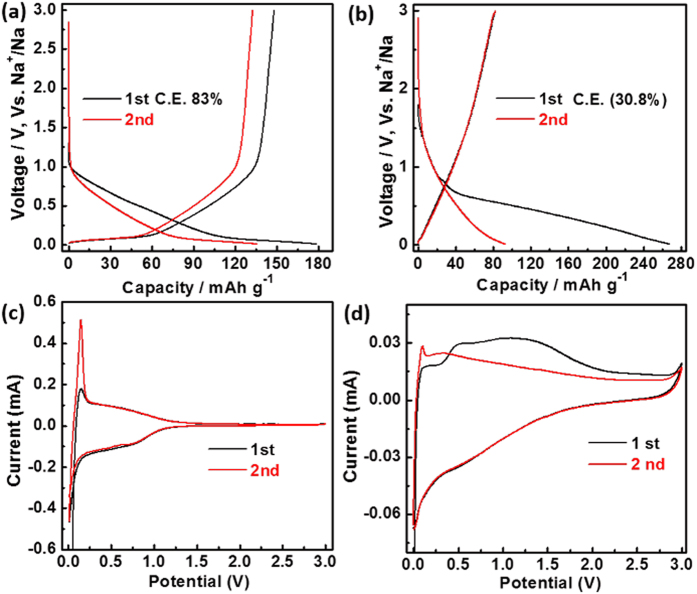
(**a,b**) Charge-discharge voltage profiles for DPC and A-DPC electrodes at a current density of 20 mA g^−1^; (**c,d**) CV curves for DPC and A-DPC electrodes at a scan rate of 0.1 mV s^−1^.

DPC and A-DPC electrodes exhibited bad performance compared with DPC-A as the anodes for NIBs (Fig. 5 and Fig. S3). Although DPC has the highest Coulomb efficiency of 83% in the first cycle, the capacity was only 179 and 136 mAh g^−1^ at 20 mA g^−1^ for the 1^st^ and 2^nd^ cycles, respectively (Fig. 5a). As revealed by XPS (Fig. S4) and IR (Fig. S5), all three samples have the same surface structure. Microscopic structure and surface area could be key reasons for the different performances. The smallest surface area is beneficial for limited side reactions, which was confirmed with the disappearance of the surplus peak in the CV curves (Fig. 5c). However, the small surface area also has a negative effect on the performance; it limits the transfer of Na ions into the electrode, and the detailed capacity contribution of the two mechanisms for the three samples is shown in Figure S6. The main discharge curves of the A-DPC electrode was the slope region, and this is the largest among the three samples for this region (Fig. S6). This suggested that storage of Na ions for nano-sheets occurred at defect sites^16^. The larger surface area of A-DPC would consume more Na ions for the formation of SEI and electrolyte decomposition, which likely led to its low first-cycle Coulomb efficiency of 36% and the observation of a large surplus peak in the CV (Fig. 5d); it also has a small plateau region owing to the small interlayer spacing^16^. DPC-A has a moderate surface area, which limited the side reactions and improved Na-ion transfer, so it had high first-cycle Coulomb efficiency and good performance.

## Conclusions

In summary, micro-nano structure hard carbon derived from filter paper was prepared by high temperature pyrolysis and further KOH activation treatment and exhibited a first-cycle Coulomb efficiency as high as 81% and a good capacity of 286 mAh g^−1^ after 100 cycles as the anode for NIBs. The excellent performance resulted from the micro-nano structural features, which reduced the first-cycle irreversible capacity loss by limiting the surface area and improving the capacity via the nano-particles. To further confirm the advantage of the micro-nano structure, a comparison of filter paper-derived bulk carbon and nano-sheet carbon was also systematically performed. The results indicate that they are inferior to micro-nano structure carbon and suggest that the micro-nano structure hard carbon is an ideal anode candidate for Na-ion batteries.

## Methods

### Materials

Filter paper was obtained from the Special Paper Industry Company (Hangzhou) and cut into small pieces. KOH was purchased from the Kemiou Chemical Reagent Company (Tianjin) and used without further purification.

### Preparation of filter paper-derived micro carbon (denoted as DPC)

Typically, 3 g of the filter paper precursor was loaded in a tubular furnace for the pyrolysis carbonization process (800 °C for 2 h, heating rate: 5 ° Cmin^−1^) under argon atmosphere. The obtained product was ground and washed with 0.1 M HCl and deionized water, then collected by filtration. After drying at 80 °C for 12 h, direct pyrolysis micro carbon (DPC) is obtained.

### Preparation of filter paper-derived micro-nano carbon (denoted as DPC-A)

Some of the DPC sample was dispersed in 7 M aqueous KOH solution and stirred for 2 hours (the mass ratio of KOH:C is 20:1), followed by another 24 hours of static soaking in ambient conditions. The extra KOH solution was removed by filtering the mixture through filter paper, and then the mixture was dried at 110 °C for 12 hours. The solid mixture was activated by KOH at 800 °C for 2 hours under argon atmosphere. After cooling down, the mixture was repeatedly washed with de-ionized water until a pH value of 7 was reached. Then, the sample was dried at 65 °C for 12 hours. Finally, filter paper-derived micro-nano carbon was obtained, which was denoted as DPC-A.

### Preparation of filter paper-derived nano-sheet carbon (denoted as A-DPC)

The synthesis process for A-DPC was the same as DPC-A, except the starting material. The starting material was original filter paper for A-DPC, while it was DPC for DPC-A.

### Material characterization

X-ray powder diffraction (XRD) patterns were acquired on a Rigaku D/MAX2200PC diffractometer with Cu Kα (λ = 1.54178 Å) radiation operated at room temperature. Raman spectra were collected on an InVia confocal Raman microscope system with the 532 nm line of an argon ion laser used as the excitation source at room temperature. X-ray photoelectron spectroscopic (XPS) measurements were performed on an X-ray photoelectron spectrometer (ESCALab MKII). The FTIR spectra (KBr pellet technique, 0.1% sample: KBr mass ratio) were recorded on a Spectrum One Perkin–Elmer Fourier Transform Instrument, averaging 100 scans at a nominal resolution of 4 cm^−1^ to improve the signal-to-noise ratio. The morphologies were characterized using field emission scanning electron microscopy (FE-SEM, Rigaku S4800) and high-resolution transmission electron microscopy (HRTEM, JEOL 2100F). The pore size distributions were calculated from the adsorption branch of the N_2_ adsorption isotherm with a Brunauer–Emmett–Teller surface area analyzer (BET, Micromeritics ASAP2020).

### Electrochemical measurements

For the electrode preparation, the derived hard carbon (80 wt%), carbon black (10 wt%, Super-P), and polyvinylidene fluoride (PVDF) binder (10 wt%) in N-methyl-2-pyrrolidone were mixed. The obtained homogenous slurry was pasted on Cu foil, followed by drying in a vacuum oven at 110 °C for 12 h. Electrochemical test cells (2032 coin cells) were assembled in an argon-filled glove box (O_2_ ≤ 0.1 ppm, H_2_O ≤ 3 ppm) with Cu foil covered by active materials as the working electrode, Na metal foil as the counter/reference electrode, and a 1 M solution of NaClO_4_ in a 1:1 vol/vol mixture of ethylene carbonate/propylene carbonate as the electrolyte. Glass fiber (Whatman) was used as a separator. The batteries were charged and discharged galvanostatically in the fixed voltage window between 0.01 V to 3.0 V on a Neware battery tester (Shenzhen, China) at room temperature. Cyclic voltammetry (CV) was carried out at room temperature on an electrochemical workstation (CHI 660E).

## Additional Information

**How to cite this article**: Zheng, P. *et al*. Micro-nano structure hard carbon as a high performance anode material for sodium-ion batteries. *Sci. Rep.* **6**, 35620; doi: 10.1038/srep35620 (2016).

## Supplementary Material

Supplementary Information
