# Supplementary material for: Micro-nano structure hard carbon as a high performance anode material for sodium-ion batteries
Source: Sci Rep. 2016 Oct 18;6:35620. doi: 10.1038/srep35620 (PMC5067775; doi:10.1038/srep35620)
Supplement: Supplementary Information [file srep35620-s1.docx]

**Supporting Materials**

**Micro-nano structure hard carbon as a high performance anode material for sodium-ion batteries**

Peng Zheng, *^a^ Ting Liu,^a^ and Shouwu Guo, *^a,b^

a School of Materials Science and Engineering, Shaanxi University of Science and Technology, Xian 710021, Shaanxi, P. R. China.

b Department of Electronic Engineering, School of Electronic Information and Electrical Engineering, Shanghai Jiao Tong University, Shanghai 200240, P. R. China

zhengpeng@sust.edu.cn; swguo@sjtu.edu.cn


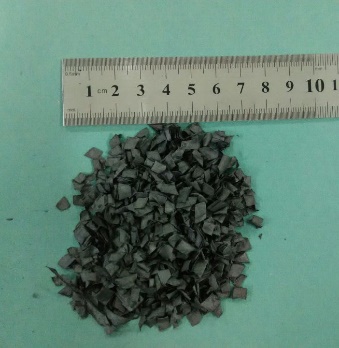


**Figure S1.** Photograph of the high temperature pyrolysis product (DPC).


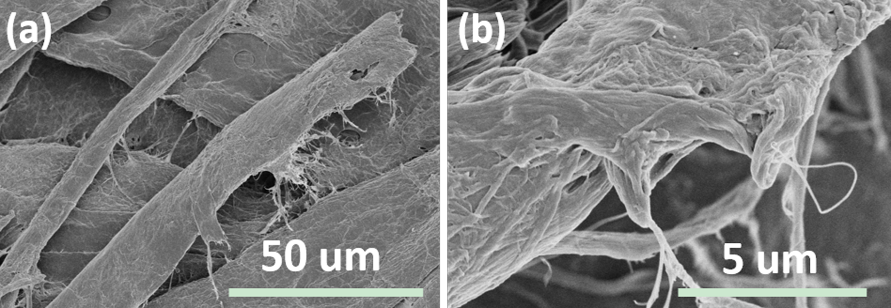


**Figure S2.** (a, b) SEM images of original filter paper.


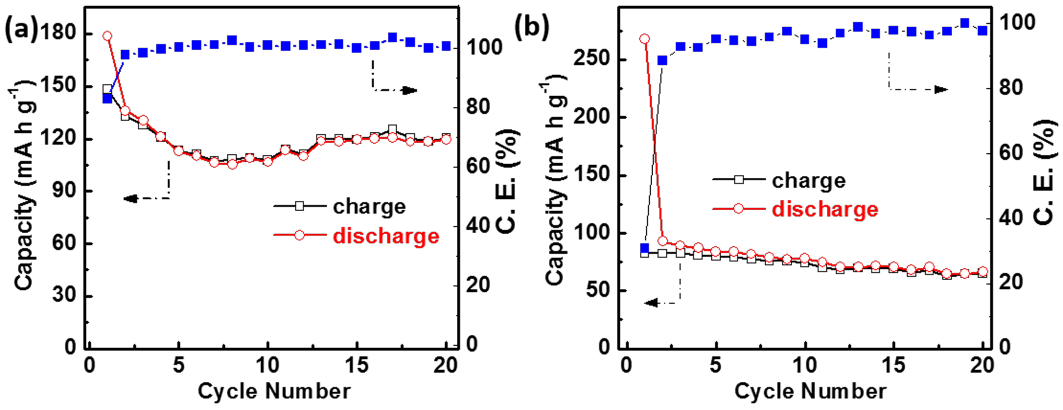


**Figure S3**. Cycling performance (circle) and the corresponding Coulombic efficiency (diamond) of the DPC electrode (a) and A-DPC electrode (b).


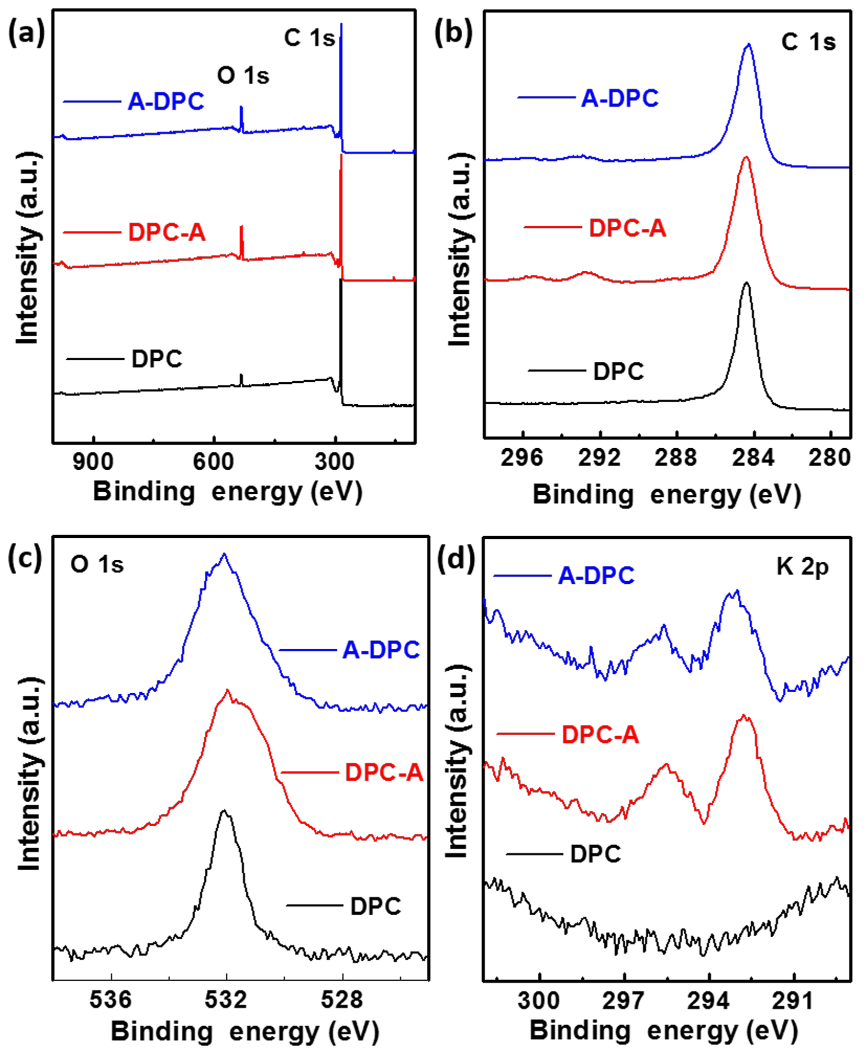


**Figure S4.** (a) XPS general spectrum, (b) C1s spectrum, (c) O1s spectrum and (d) K2p spectrum for filter paper-derived carbons.





**Figure S5**. FTIR spectra of filter paper-derived carbons.





**Figure S6**. Summary of capacity potential distribution of DPC, DPC-A and A-DPC electrodes for last cycle at current density of 20 mA g^-1^.
